# Intercorrelations of morphology with hemodynamics in intracranial aneurysms in computational fluid dynamics

**DOI:** 10.17712/nsj.2017.3.20160452

**Published:** 2017-07

**Authors:** Tianlun Qiu, Guoliang Jin, Wuqiao Bao, Haitao Lu

**Affiliations:** *From the Department of Neurosurgery (Qiu, Jin, Bao), Shaoxing People’s Hospital, Shaoxing, Zhejiang, and the Department of Neurosurgery (Lu), Chongming Brach of Shanghai Xinghua Hospital, Chongming, Shanghai, China*

## Abstract

**Objective:**

To measure morphological indices and wall shear stress (WSS) of aneurysms and parent artery surface in order to explore the relationship of morphological characteristics and WSS.

**Methods:**

Data from 47 events of consecutive cerebral saccular aneurysms from 39 patients which were referred to the interventional Neuroradiology service of the Shaoxing People’s Hospital, Shaoxing, China between 2014 April and 2015 August. Wall shear stress and wall pressure (WP) of the pre-aneurysm, aneurysm and near vessel (<1.0 cm) surface were obtained. Correlation analysis was carried between morphological parameters and WSS and its ratio. WSS, WP, intra-aneurysmal flow pattern, and location of aneurysms were analyzed.

**Results:**

Impaction zone from inflow jet was located in the distal neck part of aneurysm with high WSS in 36 aneurysms (76.6%). There were significant differences in WSS between pre-aneurysm surface and near vessel (p<0.001), aneurysm (p<0.001), aneurysm and near vessel (p<0.001). Significant correlations were found between aneurysm WSS and aspect ratio (r=-0.296), aneurysm-artery WSS ratio and size ratio (r=-0.322), aspect ratio (r=-0.416).

**Conclusion:**

Uneven WSS distributes in the various part of the pre-aneurysm vessel. The impaction zone from inflow jet is located in the distal neck of aneurysm. Aspect and size ratios can effect aneurysm WSS

Intracranial aneurysm is a pathological embossment of the artery wall due to local expansion. Hemodynamics play a critical role in the initiation, development, and rupture of aneurysms,^1^,^2^ but there is no exact evidence that aneurysms rupture because of blood pressure on the artery wall. Hemodynamic parameters include wall shear stress (WSS), wall pressure (WP), aneurysm vortex, and blood flow velocity, but their relation to aneurysm rupture remains inconsistent and confusing.^3^-^5^ The most highlighted and controversial parameter is WSS, which is the frictional force exerted by the flowing blood tangentially on the vessel lumen. Both high and low aneurysmal WSS have been correlated with intracranial aneurysm growth and rupture.^4^ Elevated WSS caused by the impaction of a concentrated inflow jet may have a damaging effect on the arterial wall, which could cause aneurysm formation, at least based on in vitro and in vivo studies,^6^-^9^ but studies are limited in humans by difficulties measuring hemodynamics parameters before aneurysm occurrence. Previous studies used computational models of cerebral aneurysms from 3D rotational angiographies and found that most blebs occurred at or adjacent to the aneurysm region with the highest WSS before bleb formation, near the flow impaction zone.^10^-^12^ It is presumed that the final shape of the aneurysm is determined by the interaction of a flow-induced wall injury, the biomechanical response of the wall, and the interaction with extravascular structures of the peri-aneurysmal environment,^6^ but how peri-aneurysmal structures affect fluid dynamics after they occur is unclear and remains to be clearly elucidated. Therefore, the aim of the present study was to measure morphological indices and WSS of aneurysms and parent artery surface in order to explore the relationship of morphological characteristics and WSS. The strength differences of WSS among various aneurysms were also assessed to explore the role of WSS in different development stages of aneurysms. The results of the present study could provide new insights about the formation of aneurysms and could play a role in the prevention and treatment of aneurysms.

## Methods

### Patients

Data from 47 events of consecutive cerebral saccular aneurysms from 39 patients were included in this study. The patients were referred to the Interventional Neuroradiology Service of the Shaoxing People’s Hospital between 2014 April and 2015 August and diagnosed with cerebral aneurysms by conventional catheter angiography and digital subtraction angiography (DSA).

### Imaging

Rotational angiography images were obtained during a 180° rotation and imaging at 15 frames per second for a total of 8 s, using an Integris system (Philips Medical Systems, Best, The Netherlands). The corresponding 120 projection images were reconstructed into a three-dimensional data set of 128×128×128 voxels covering a field of view of 54.02 mm on a dedicated Philips workstation.

### Two-dimensional morphological assessments of aneurysms

**Figure 1:**
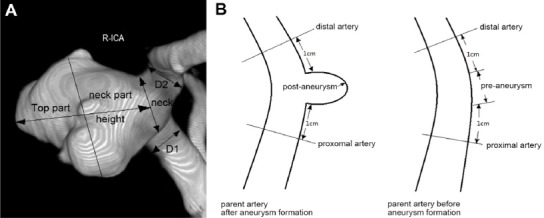
Two-dimensional morphological assessments of aneurysms **A)** The longest dimension from the center of the neck to the dome tip was the height of the aneurysm (Hmax). The aneurysm was divided into top and neck parts, half and half, according to Hmax and the parting line parallel to the neck plane. The average value of both sides of the aneurysm neck was D1+D2/2 (average parent artery diameter). Aspect ratio (height/Dneck) and size ratio (height /Dparent) are considered as the 2 main morphological indices. **B)** Hemodynamics parameters of vessel before and after aneurysm formation.

Two-dimensional (2D) variables were measured by DSA: height (the maximum distance between the center of the aneurysm neck and the dome of the aneurysm), average neck diameter (Dneck), average parent artery diameter (Dparent) (**Figure 1**). Measurements were not necessarily in the same plane, but planes were selected in which all 2D measurements were in the same plane to explain. Based on these measurements, 2D indices were calculated: aspect ratio (height/Dneck) and size ratio (height/Dparent).

### Computational fluid dynamics

**Figure 2:**
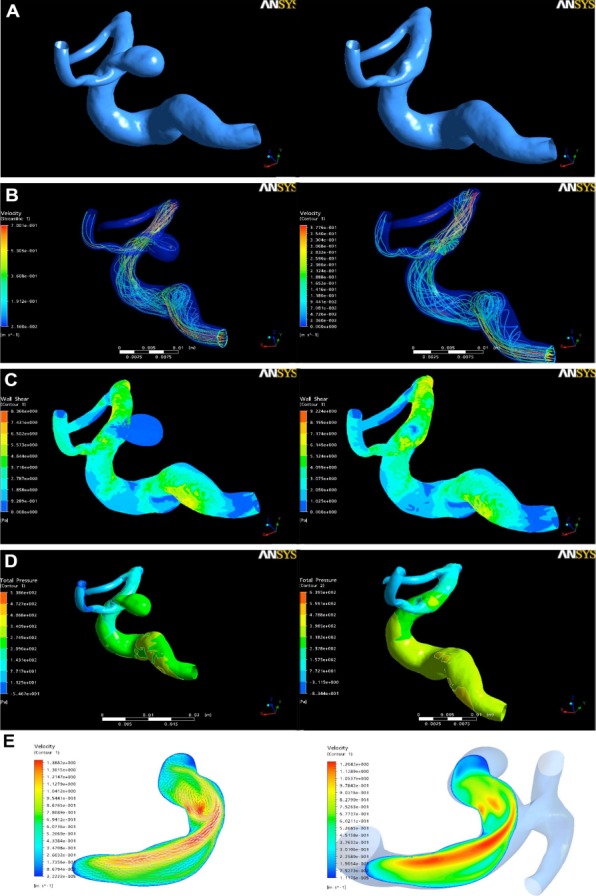
Modeling of aneurysms and hemodynamics analysis **A)** Parent artery model before and after aneurysm, **B)** Streamline distribution in different parts of the vessel before and after aneurysm formation, **C)** Wall shear stress (WSS) distribution in different parts of the vessel before and after aneurysm formation, **D)** Wall pressure (WP) in different parts of the vessel before and after aneurysm formation, **E)** Streamline velocity diffusion in parent artery and aneurysm.

DSA image was constructed for each patient by removing the aneurysm with GEOMAGIC studio 12 (Geomagic, Cary, NC, USA). This was achieved by cutting the aneurysms off and smoothing the surface of the aneurysm region, as manually delineated on the original anatomic model. The final model without the aneurysm was denoted as the “vessel before aneurysm formation,” whereas the original vessel harboring the aneurysm is denoted as the “vessel after aneurysm formation.” (**Figure 2 A-B**)

The area of pre-aneurysm surface in the vessel before aneurysm formation is equal to aneurysm neck area. The parent vessel <1.0 cm away from the edge of the pre-aneurysm area was called “near parent vessel”. Each model was meshed and created for each patient to analyze the vessel before and after aneurysm formation (ANSYS Inc., Canonsburg, PA, USA). Pulsatile blood flow simulation was carried out by numerically solving the 3D Navier-Stokes equations for a Newtonian incompressible fluid with a density of 1056 kg/m^3^ and a viscosity of 0.0035 N·s/m^2^ under the assumption of rigid vessel walls and no slip boundary conditions. Pulsatile flow conditions were introduced as inflow conditions in the internal carotid (ICA), with an average flow set at 0.254 L/min and pulse at 70 bpm. Fully developed pulsatile velocity profiles were prescribed using the Womersley solution with Navier-Stokes equations.^13^-^15^ Traction-free boundary conditions with the same pressure level were applied at outlet boundaries. The average Reynolds number was within the range of normal blood flow for human cerebral arteries,^16^ indicating a laminar flow condition.^17^ A total of 800 time steps (0.001 s/step) were set for each cardiac cycle.

### Modeling of aneurysms and hemodynamics analysis

The mean WSS and WP of near vessel (1.0 cm away from pre-aneurysm surface) and pre-aneurysm surface in a cardiac cycle were measured in all 47 events. The mean WSS of aneurysm and near parent vessel, and the mean WP of aneurysm were measured in all 47 events. Aneurysm-artery WSS ratios (the ratio of WSS between aneurysm and near parent artery) were calculated to assess fluid characteristics of aneurysms and parent arteries. Hemodynamics characteristics included WSS and WP. WSS and WP distribution was examined in a vessel before and after aneurysm formation (**Figure 2C-D**). The injection flow area and impact area, and their position in velocity streamline picture of longitudinal profile impact area were observed after the aneurysm was formed (**Figure 2E**).

### Statistical analysis

Statistical analysis was performed using SPSS 16.0 (IBM, Armonk, NY, USA). The Smirnov-Kolmogorov test was performed to determine the normality distribution of continuous variables. Normally distributed continuous variable are expressed as mean ± standard distribution and were analyzed using the paired sample t-test. Non-normally distributed variables are presented as median and quartiles, and were analyzed using the Wilcoxon rank-sum test. Correlation analyses between hemodynamic parameters including WSS of aneurysm, aneurysm-artery WSS ratio, and geometrical morphology parameters (size ratio and aspect ratio) were performed to explore how geometrical morphology effect hemodynamic characteristics. Two-sided *p*-values <0.05 were considered statistically significant.

## Results

### Characteristics of the aneurysms

Among the 39 patients, 32 were found to have one aneurysm, 6 had 2 aneurysms, and one had three aneurysms. The location of these aneurysms was: 29 in the ICA, nine in the middle cerebral artery, five in the basilar artery, 3 in the internal carotid artery terminus, and one in the anterior cerebral artery (anterior cerebral artery-pericallosa). There were 36 ruptured and 11 unruptured aneurysms

### Hemodynamics

After aneurysm formation, the velocity streamline picture of the longitudinal profile shows that the impact area of the injection flow always lies in the distal neck part of the aneurysm with high WSS in 36 aneurysms (76.6%) (**Figure 2E**).

The WSS and WP of the pre-aneurysm surface and near parent vessel were recorded. The mean WSS was 7.43±3.83 Pa on the near parent vessel surface before aneurysm, 9.92±5.58 Pa on the pre-aneurysm area, 5.18±3.84 Pa on the aneurysm surface, and 7.87±4.76 Pa on the near vessel surface after aneurysm. The aneurysm-artery WSS ratio was 0.67±0.27, the aspect-ratio was 1.62±0.84, and the size ratio was 1.98±1.13 (**Table 1**). wall pressure of the near vessel surface before aneurysm was 862 (IQR: 406) Pa, pre-aneurysm surface was 880 (IQR: 547) Pa, and aneurysm surface was 902 (IQR: 475) Pa (**Table 2**).

**Table 1:** Wall shear stress of the different parts of the artery before and after aneurysm formation.

Variables	Near vessel surface before aneurysm	Pre-aneurysm surface	Aneurysm surface	Near vessel surface after aneurysm
WSS (Pa)	7.43±3.83	9.92±5.58	5.18±3.84	7.87±4.76
Variable	Aneurysm-artery WSS ratio	Aspect ratio	Size ratio	
Ratio	0.67±0.27	1.62±0.84	1.98±1.13	

WSS - wall shear stress

**Table 2:** Wall pressure of the different parts of the artery before and after aneurysm formation.

Variables	Near vessel surface before aneurysm	Pre-aneurysm surface	Aneurysm surface
***WP (Pa)***			
Median	862.38	880.01	902.32
Inter-quartile range	405.53	547.03	474.98

WP - wall pressure

Paired comparisons were performed for WSS and WP among different parts of vessel before and after aneurysm. There were significant differences in WSS between pre-aneurysm surface and near vessel surface before aneurysm (*p*<0.001), pre-aneurysm surface and aneurysm surface (*p*<0.001), and aneurysm surface and near vessel surface after aneurysm (*p*<0.001), but there was no difference for WP between pre-aneurysm surface and near vessel surface before aneurysm (*p*=0.649), and pre-aneurysm surface and aneurysm surface (*p*=0.719) (**Table 3**).

**Table 3:** Paired comparisons for WSS and WP of different parts of vessel before and after aneurysm formation.

Paired comparison	Pre-aneurysm surface and near vessel before aneurysm	Pre-aneurysm surface and aneurysm surface	Aneurysm surface and Near vessel after aneurysm
WSS	<0.001*	<0.001*	<0.001*
WP	0.649†	0.719†	

WSS - wall shear stress, WP - wall pressure,

* t-test,

† Wilcoxon rank-sum test

**Figure 3:**
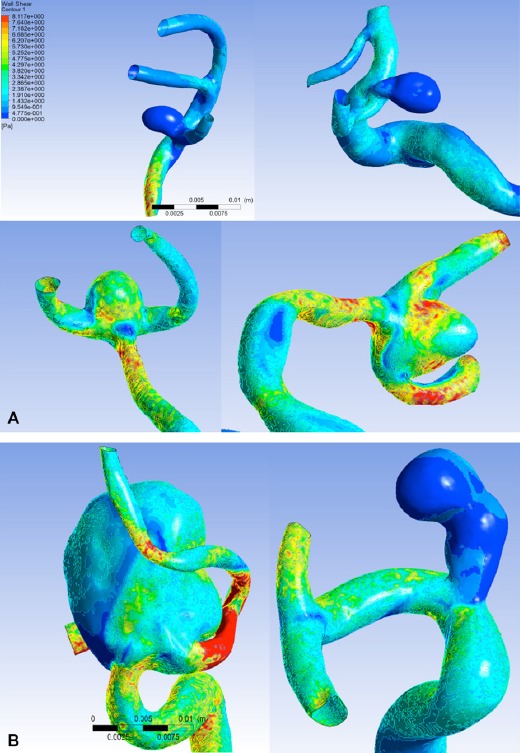
Morphological effect on wall shear stress in intracranial aneurysms **A)** Wall shear stress (WSS) of the aneurysm. Red represents higher WSS, blue represents lower WSS. There was a negative significant correlation between aneurysm WSS and aspect ratio (height/neck diameter). Aneurysm WSS is high under low aspect ratio. On the contrary, high aspect ratio result in low aneurysm WSS. **B)** Although size ratio (height/diameter of the parent artery) are both high in the 2 figures, the resulting WSS is not similar, suggesting that aneurysm WSS and size ratio are not significantly correlated.

Correlation analyses between hemodynamic parameters and geometrical morphology parameters were performed. Significant negative correlations were found between aneurysm WSS and aspect ratio (r=-0.296), aneurysm-artery WSS ratio and size ratio (r=-0.322), and aneurysm-artery WSS ratio and aspect ratio (r=-0.416). There was no significant correlation between aneurysm WSS and size ratio (**Table 4** and **Figure 3**).

**Table 4:** Correlation analyses between hemodynamic and geometrical morphology variables.

Variables	Aneurysm WSS and aspect ratio	Aneurysm WSS and size ratio	Aneurysm-artery WSS ratio and size ratio	Aneurysm-artery WSS ratio and aspect ratio
Pearson r	-0.296^*^	-0.17	-0.322^*^	-0.416^**^
*P*-value	0.043	0.253	0.028	0.004

* significant correlation at *p*<0.05,

** significant correlation at *p*<0.01, WSS - wall shear stress

## Discussion

The present study showed that the impaction zone from inflow jet was located in the distal neck part of aneurysm with high WSS in 36 patients (76.6%). Wall shear stress was distributed heterogeneously along the parent artery wall. Pre-aneurysm surface WSS was higher than near parent artery before aneurysm formation. This unbalanced diffusion of WSS could be ascribed to morphological restriction in the structural cranial base. The WSS is sensed by endothelial cells, which transduce this mechanical signal into biological signals, activating biochemical pathways that maintain vascular homeostasis and regulate remodeling.^18^ Aberrant WSS elicit endothelial cell-mediated pathological responses, including pro-inflammatory behavior,^19^ matrix metalloproteinase activation,^20^ cell death,^21^ extracellular matrix degradation, and destructive remodeling.^8^,^22^,^23^ Metaxa et al^8^ demonstrated that aneurysm initiation occurs when high WSS and positive WSS gradient exceed a certain threshold. This insult leads to local internal elastic lamina loss, media thinning, and bulge formation.^7^,^8^,^23^ In the present study, the high WSS area was the same as the area the aneurysm neck lied in, which is in accordance with the high WSS theory. These results suggest that correlating aneurysm location to a maximum, and not simply a region of predefined high WSS, has the potential advantage of providing increased accuracy in prospective predictive models by limiting the search area for future aneurysm formation.

After the aneurysm initiation phase (which has been determined to be driven by high WSS and high, positive WSS gradient,^8^,^9^ aneurysm sac enlargement lead to low WSS,^24^ which is explained by the presence of a countercurrent vortex within the aneurysm region. In the 47 models studied here, the major flow structures were not affected by the presence or absence of the aneurysm; however, small changes within the parent artery could alter the relative position of the aneurysm and thus change its hemodynamic relationships. We do acknowledge that there are many assumptions in this model that need to be further validated.

An in vitro experiment demonstrated that the degeneration of endothelial cells occurred due to the exposure of vessel endothelium to low WSS (<0.4 Pa).^25^ Boussel et al^2^ analyzed seven growing aneurysms in a follow-up study and found a linear correlation between the local displacement of the intracranial aneurysms wall (that is, enlargement) and low WSS. The low flow theory points to localized stagnation of blood flow against the wall in the dome as causing a dysfunction of the endothelium.^21^,^26^,^27^ On the other hand, the distinguishing feature between impinging flow may persist after bulge formation in some aneurysms, so that high WSS and positive WSS gradient could remain prevalent in the aneurysmal sac.^23^,^28^-^30^ A recent study showed that pressure difference was an important parameter for thin-walled regions of aneurysms.^31^

Taken together, high WSS and low WSS are 2 aberrant hemodynamic conditions that could elicit pathological remodeling pathways to drive intracranial aneurysm growth and rupture. In morphological structure, size ratio will increase due to developing aneurysm after initiation, as well as the aspect ratio.^32^ In the present study, WSS of the aneurysm area was lower than that of pre-aneurysm and parent artery, because hemodynamic models change after aneurysm occur and because aneurysm WSS increases its aspect ratio. Inlet blood flow reduces with increasing aspect ratio, which leads to low-level WSS. Inlet blood flow reduces with increasing size ratio relative to aneurysm, but neck width increases with size ratio in some aneurysms, which lead to relatively more inlet flow in the aneurysm. Therefore, neck width could be a confusing factor in aneurysm development. Size ratio is only partially related to aneurysm WSS. Various magnitude and formation of parent artery could result in diverse inlet blood flow. The aneurysm-artery WSS ratio could be a more sensitive variable to show hemodynamic changes than aneurysm WSS only.

Aneurysmal geometry and hemodynamics are mutually causal.^33^ Geometry determines flow conditions, while flow drives aneurysm remodeling/growth, thereby determining geometry (i.e., enlargement and shape change).^26^ The WSS distribution in the aneurysm changes with the growing aneurysm. In the present study, the inlet flow often inject near the distal wall of aneurysm neck part, and high WSS is always found in small aneurysms and it often appears in near distal wall of the aneurysm neck part, which illustrates that high WSS is an important factor for aneurysm development in the early stage. Low WSS then become a main factor along with growing aneurysm. This hemodynamic change could be a gradual course of interactions between morphological and hemodynamics with aneurysm formation and growth, but longitudinal studies are necessary to ascertain this point. Shojima et al^34^ studied 29 aneurysms in 26 patients and found that WP was increased in curved arteries. Blood flow enters into aneurysms with decreasing velocity in curves and bifurcations. The velocity at the rupture point and dome of aneurysms are lower than in the parent artery, and the impact force in the aneurysm is weak.^34^ In the present study, results indicate that there was no significant difference between pre-aneurysm surface and aneurysm, which strongly suggest that blood flow impact and partial pressure do not obviously lead to aneurysm rupture and growth alone. The WP of pre-aneurysm is a little higher than near parent vessel surface before aneurysm formation, but it there was no significant difference. Blood pressure elicits tensile stresses in the wall, which, when unbalanced, stimulate collagen synthesis, cross-linking, and degradation.^35^ Wall pressure could contribute to aneurysm formation not only through WSS but also through kinetic energy on the artery wall for a long time. Many factors are involved in the formation of aneurysms and additional studies are necessary to understand their relationships.^36^

The present study is not without limitations. The modeling itself probably introduces a bias since it is difficult to smooth the artery surface precisely when removing the aneurysm using the GEOMAGIC software. The impact area of injection flow is imprecise in view of absent reasonable geometric division. Finally, some assumptions like assuming that the blood vessel has a rigid arterial wall structure and that the blood is a Newtonian fluid introduce additional biases.

In conclusion, dynamic interactions between hemodynamics and aneurysm morphology may play critical roles in the development and growth of aneurysms. Additional studies using more refined models are still necessary to improve our knowledge of aneurysm formation.
